# Supplementary material for: Dose-Ranging Plasma and Genital Tissue Pharmacokinetics and Biodegradation of Ultra-Long-Acting Cabotegravir In Situ Forming Implant
Source: Pharmaceutics. 2023 May 13;15(5):1487. doi: 10.3390/pharmaceutics15051487 (PMC10222572; doi:10.3390/pharmaceutics15051487)
Supplement: Supplementary file 1 [file pharmaceutics-15-01487-s001.zip › pharmaceutics-2352262-supplementary.pdf]

pharmaceutics

MDPI

Supplementary Materials: Dose-ranging plasma and genital tissue pharmacokinetics and biodegradation of ultra-long-acting cabotegravir in situ forming implant

Isabella C. Young, Allison Thorson, Roopali Shrivastava, Craig Sykes, Amanda Schauer, Mackenzie Cottrell, Angela D.M. Kashuba, S. Rahima Benhabbour

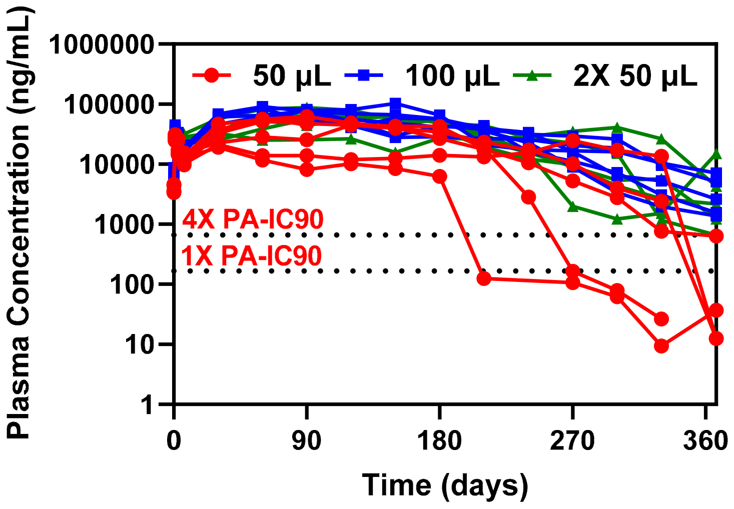

Figure S1. Individual replicates of CAB plasma concentrations after 367 days post-injection. CAB plasma concentrations after 50 µL, 100 µL, or 2×50 µL injection of CAB ISFI for 367 days. 4× PA-IC90 is 664 ng/mL and 1× PA-IC90 is 166 ng/mL. Each dose elicited n=5-6 mice/timepoint.

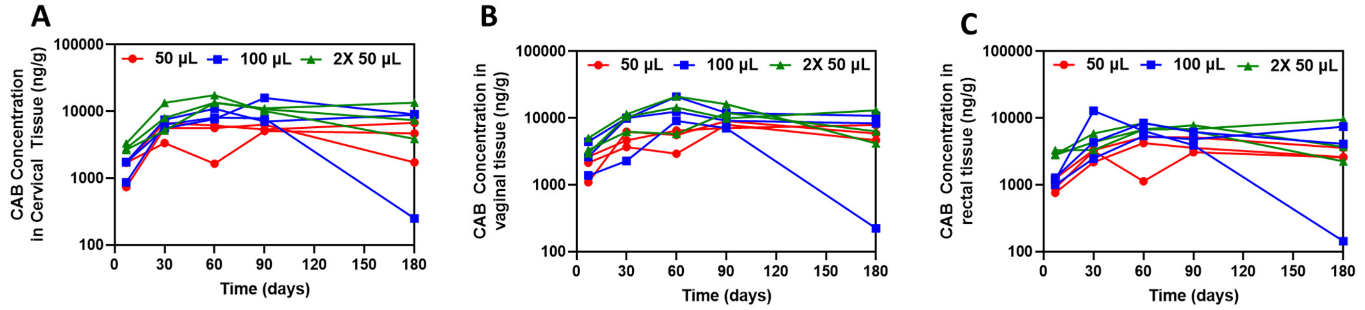

Figure S2. Individual replicates of CAB concentrations in tissues. (A) Individual replicates (n = 3/timepoint per dose) of CAB concentration in cervical tissue after CAB ISFI injection in female BALB/c mice. (B) Individual replicates (n = 3/timepoint per dose of CAB concentration in vaginal tissue after CAB ISFI injection in female BALB/c mice. (C) Individual replicates (n = 3/timepoint per dose) of CAB concentration in rectal tissue after CAB ISFI injection in female BALB/c mice.

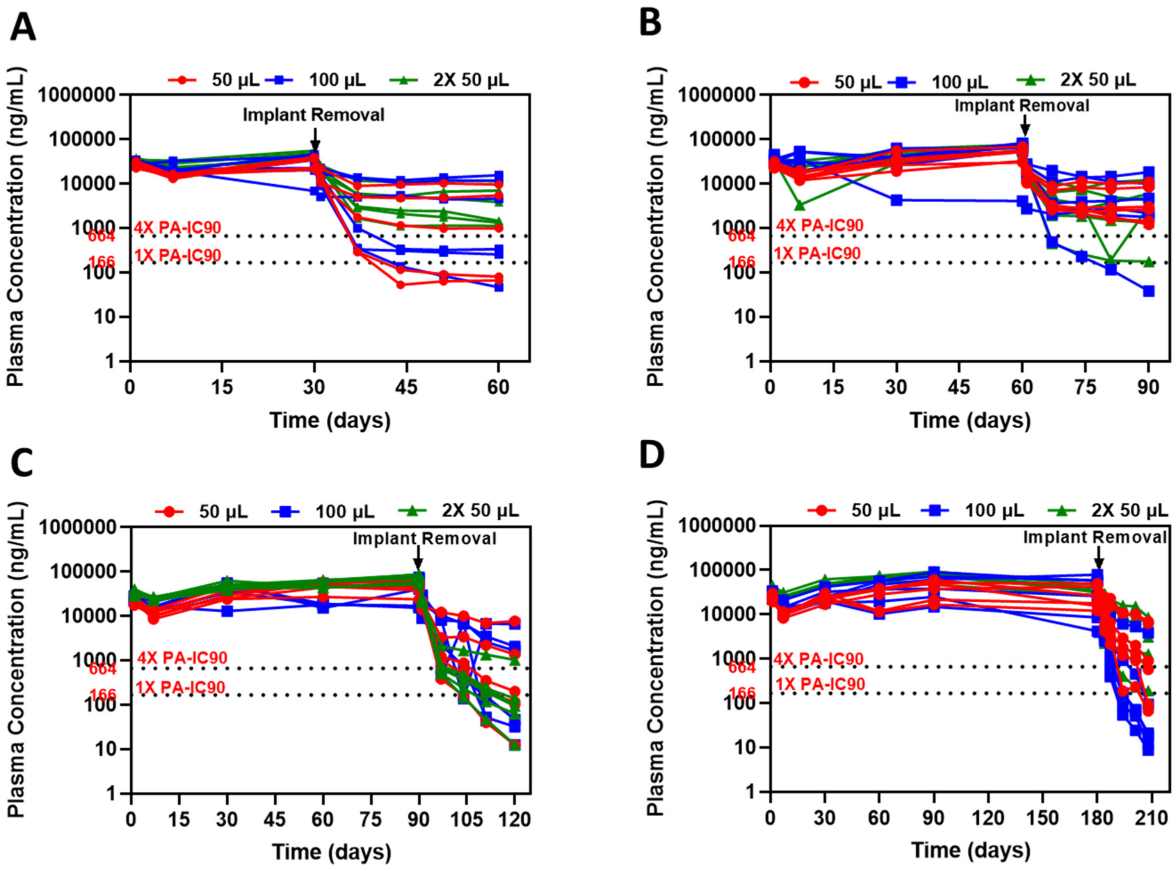

Figure S3. Individual replicates of CAB plasma concentrations after depot removal. (A–D) Individual replicates (n = 5–6/timepoint per dose) of CAB concentrations in plasma after ISFI removal at 30, 60, 90, and 180 days post-administration, respectively.
